# Therapeutic Potential of Select Berry Varieties in Mitigating Cognitive Decline via Oxidative Stress and Insulin Resistance in AlCl_3_‐Exposed Mice

**DOI:** 10.1002/brb3.71411

**Published:** 2026-04-21

**Authors:** Sawsan G. A. A. Mohammed, M. Walid Qoronfleh, Reem Al Alawi, Shadhan Al‐Siyabi

**Affiliations:** ^1^ Clinical Science Department, QU Health, College of Medicine Qatar University Doha Qatar; ^2^ Healix Lab Muscat Oman; ^3^ Healthcare Research & Policy Division Q3 Research Institute (QRI) Ann Arbor Michigan USA; ^4^ Department of Chemistry, College of Science Sultan Qaboos University Muscat Oman; ^5^ Department of Biomedical Science, College of Medicine and Health Sciences Sultan Qaboos University Muscat Oman

**Keywords:** aging, AlCl_3_, Alzheimer's disease, berries, insulin resistance, memory, neuropharmacology, neuroprotection, oxidative stress

## Abstract

**Purpose:**

Oxidative stress damage and impaired insulin receptor (IR) signaling are critical risk factors for contributing to cognitive impairment in Alzheimer's disease (AD) and other neurological disorders. The beta‐amyloid peptide (Aβ) oligomer deposition in the hippocampal region decreases the IR expression. This study aimed to assess whether berries grown in Oman (*Morus alba*, *Morus macroura*, and *Sideroxylon mascatense*) could ameliorate dysfunction of the antioxidant system and restore the IR expression thereby improve the cognitive performance in aluminum chloride (AlCl_3_) induced AD mouse model.

**Methods:**

Various biochemical assays were conducted to estimate antioxidant capacity. The total phenolic (TPC) and flavonoid (TFC) contents for the three varieties were quantified using Folin–Ciocalteu and AlCl_3_ calorimetric methods, respectively. The antioxidant properties of the three berries were analyzed using thiobarbituric acid reactive substances assay (TBARS), superoxide dismutase assay (SOD), glutathione assay (GSH), glutathione peroxidase assay (GPx), and estimation of glutathione reductase (GR). Cognitive performance was measured using Morris water maze (MWM) and T‐maze.

**Findings:**

Berries, particularly *M. alba* and *S. mascatense*, demonstrated the highest phenolic and flavonoid content and exhibited strong antioxidant activity across all assays. Mice fed with *M. alba*, *M. macroura*, and *S. mascatense* showed better performance parameters in both MWM (spatial learning and memory) and T‐maze (time taken to reach the platform) tests, along with enhanced IR signaling (*p *< 0.01).

**Conclusion:**

These findings suggest that supplementation with mulberries may improve cognitive decline and insulin resistance induced by AlCl_3_, highlighting their potential as neuroprotective agents in AD.

## Graphical abstract

Select berries are rich in flavonoids and polyphenols and have potent free radical scavenging and neuroprotective properties. They also prevent aggregation of Aβ and anti‐glycation substances, and thereby exhibit neuroprotective properties. Berries supplementation in AlCl_3_ fed mouse model improved overall oxidative stress parameters, spatial learning and memory measures, and upregulated insulin receptor signaling the IRS‐1 and IRS‐2 expression.

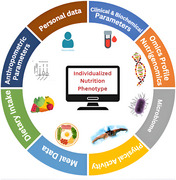

**Abbreviations**
AD: Alzheimer's disease
Aβ: amyloid beta
CAT: catalase
CEQ: catechin equivalents
GAE: gallic acid equivalents
GPx: glutathione peroxidase
GR: glutathione reductase
GSH: glutathione assay
h: hours
IR: insulin receptor
min: minutes
MWM: Morris water maze
s: seconds
SOD: superoxide dismutase assay
TBARS: thiobarbituric acid reactive substances assay
TFC: total flavonoid content
TPC: total phenolic content
WB: western blot

## 1 Introduction

Alzheimer's disease (AD) is the most prevalent cause of major neurocognitive disorders resulting from age‐dependent chronic neurodegeneration in brain regions critical for higher functioning and meaningful existence (Upadhyay et al. 2014; Bishir et al. 2020). To date, there are 56 million AD patients across the globe, and it has been projected to reach 88 million by 2050 due to significant population aging (2020 Alzheimer's Disease Facts and Figures 2020). The major molecular mechanisms and pathways involved in the development of AD have not yet been elucidated; however, aging is considered to be the prominent risk factor in AD. Epidemiological studies have revealed a temporal relationship between type 2 diabetes mellitus (T2DM), characterized by peripheral insulin resistance, and AD (Arnold et al. 2018). Clinical data have strongly implicated the progression of T2DM and the onset of neurodegenerative disorders, including AD (Kopf and Frölich 2009; Sims‐Robinson et al. 2010). Insulin resistance has also been observed in the central nervous system of AD patients (Biessels and Reagan 2015).

Recent studies in neuronal cell lines have indicated a possible link between amyloid‐beta (Aβ) metabolism and the insulin pathway (Gasparini et al. 2001; Solano et al. 2000). A drastic decrease in the neuronal surface insulin receptors (IRs) was observed following the administration of Aβ oligomers into the hippocampal region (Zhao et al. 2008; De Felice et al. 2009). The Aβ oligomer administration showed elevated levels of IRs in the cell body, promoting the internalization of IRs. The above finding was supported by Western blot (WB) assay, which revealed no change in the total number of IRs following Aβ administration (Zhao et al. 2008). Aβ administration also downregulated the *N*‐methyl‐d‐aspartate (NMDA) receptor through casein kinase 2 (CK2 or CK‐II) and Ca^2+^/calmodulin‐dependent kinase II (CaMKII) (Chung et al. 2004). Studies by De Felice et al. revealed a drastic down‐regulation of membrane surface IRs following Aβ administration, mediated through CK2 and CaMKII pathways (PNAS 2009). IRs play a crucial role in learning, memory, and tau phosphorylation; hence, Aβ induced downregulation of IRs could lead to dementia and other features of AD. Presently, there is little evidence to suggest there are effective drugs to modulate the trajectories of AD and T2DM. Therefore, there is merit to expand the role of nutrition.

Oman's natural and geographical features permit white mulberries to grow abundantly and be consumed locally. It is a preferred favored fruit by locals. Mulberries have many health benefits as they possess anti‐inflammatory properties. Berries like white mulberries (*Morus alba*) and (*Morus macroura*) and wild blueberry (*Sideroxylon mascatense*) are rich in flavonoids and polyphenols and have potent free radical scavenging and neuroprotective properties (Kobus‐Cisowska et al. 2020; Subash et al. 2014; Al Hasani et al. 2021; Kang et al. 2006). The health benefits of berries have been elaborated on by other researchers (Mohammed and Qoronfleh 2020; Essa et al. 2023). Berries also prevent aggregation of Aβ and anti‐glycation substances and thereby exhibit neuroprotective properties. Additionally, polyphenols upregulate the IRS‐1/PI3K/Akt pathway, downregulate the GSK‐3β pathway, and inhibit tau phosphorylation (Tang et al. 2018). Blueberry improved IR and glucose tolerance in T2DM (Elks et al. 2015; Nair et al. 2014). Clinical studies showed that blueberry improved insulin sensitivity in T2DM patients who consumed smoothies supplemented with berries compared to other counterparts who consumed placebo (Youdim et al. 2000). Moreover, Seymour et al. (2011) reported an increase in the number of mRNA transcripts associated with insulin receptor substrate‐1 (IRS‐1) and glucose transporter 4 (GLUT4) following consumption of blueberries for 12 weeks. A highly important, relevant studies and work in support of this investigation is that of Sood et al. (2023, 2024). They reported that *M. alba* fruit extract and diet ameliorates cognitive deficit in mouse model of streptozotocin‐induced memory impairment (Sood et al. 2023; Bazzari et al. 2019) Taking this into consideration and since *M. alba*, *M. macroura*, and *S. mascatense* are very rich in flavonoids and polyphenols; therefore, the present study was designed to understand the neuroprotective effect of the local Omani berries against AlCl_3_ induced AD‐like pathology that also simultaneously cause significant damage to the insulin signaling pathway (Bazzari et al. 2019). Thus, within the aforementioned link between AD and T2DM, the present study embarked to explore the role of berries in upregulating the IRS‐1 receptors in AlCl_3_ induced AD mice. The implicit aim of the present study is to examine the protective effects and the role of nutrition in modulating AD and T2DM.

## 2 Materials and Methods

### 2.1 Plants

The Oman berries used in this study were *M. alba* which is commonly known as the White Mulberry, *M. macroura* which is known as the King White Mulberry, Long Mulberry, or Himalayan Mulberry (both belong to Moraceae family), and *S. mascatense* (family Sapotaceae) that is commonly referred to in Oman as “būt” (pronounced “boot”) or Omani wild blueberry. In terms of market availability, in Oman, the fruiting season for both *M. alba* and *M. macroura* generally occurs during the spring to early summer, typically starting around March and peaking in April to May. Although the peak season for *S. mascatense* occurs during the summer, specifically from May to August. The source for the fruits was the fresh local market. Berries were dried by means of standard fruit freeze‐dry protocol to generate a powder to mix with ground mice feed (Nowak and Jakubczyk 2020).

### 2.2 Animals

In the present study, 64 adult female CD‐1 mice of weight 26–28 g were procured from the small animal house, Sultan Qaboos University (SQU), Oman. The sample size is based on prior conventional published experimental protocols. Animals were housed in polypropylene cages in the standard laboratory room conditions at 23°C ± 2°C, with 12/12 h light/dark cycle, the relative humidity of 55% ± 10%, and maintained with free access to chow diet and tap water (Reshma et al. 2022). For the treatment groups, 4% of mulberry and blueberry were administered by mixing it with a regular diet (20 g of the dried fruit powder was mixed with 480 g of standard chow diet). Animals were acclimatized 7 days prior to the start of the experimental procedure. Euthanasia involved CO_2_ inhalation in a special chamber followed by a physical method (decapitation) to ensure death. The study protocol was approved by SQU Animal Ethical Committee (SQU/AEC/2016‐17/5).

### 2.3 Chemical Treatment

Aluminum chloride (AlCl_3_—obtained from Sigma) exposure in mice mimics AD‐pathology. AlCl_3_ primarily accumulates in the hippocampus and frontal cortex of the brain. As a neurotoxin, it often deposits in these areas, causing oxidative stress, β‐amyloid plaque formation, and tau neurofibrillary tangles.

**Table:** 

Group	Protocol treatment	Designation
I	Saline (100 mg/kg p.o.)	Negative control (normal)
II	AlCl_3_ (100 mg/kg p.o.)	Positive control (AD pathology)
III	AlCl_3_ + 4% *Morus alba*	White mulberry treatment
IV	AlCl_3_ + 4% *Morus macroura*	White mulberry treatment
V	AlCl_3_ + 4% *Sideroxylon mascatense*	Blueberry treatment
VI	Saline + 4% *M. alba*	White mulberry alone—internal negative control
VII	Saline + 4% *M. macroura*	White mulberry alone—internal negative control
VIII	Saline + 4% *S. mascatense*	Blueberry alone—internal negative control

Abbreviations: AD, Alzheimer's disease.

### 2.4 Experimental Design and Treatment Protocol

Prior to acclimatization, animals were randomized to eight different groups with each group containing eight animals. **Group I** mice were administered with normal saline—100 mg/kg p.o. (negative control, i.e., Saline Control), **group II** animals received AlCl_3_ (100 mg/kg p.o.) (positive control, i.e., AD), **group III** animals were administered with AlCl_3_ (100 mg/kg p.o.) + 4% white mulberry (*M. alba*) powder mixed with ground mice feed, **group IV** animals were administered with AlCl_3_ (100 mg/kg p.o.) + 4% white mulberry (*M. macroura*) powder mixed with ground mice feed, **group V** animals were administered with AlCl_3_ (100 mg/kg p.o.) + 4% blueberry (*S. mascatense*) powder mixed with ground mice feed, **group VI** animals were administered with saline (100 mg/kg p.o.) + 4% white mulberry (*M. alba*) powder mixed with ground mice feed, **group VII** animals were treated with saline (100 mg/kg p.o.) + 4% white mulberry (*M. macroura*) powder mixed with ground mice feed, and **group VIII** animals were administered with saline + 4% blueberry (*S. mascatense*) powder mixed with ground mice feed. Groups VI–VIII are another set of negative control for berries. All the treatments were given for 6 weeks daily for AlCl_3_ to establish significant neurodegeneration and cognitive impairment, and for berries treated groups to exhibit neuroprotection. The 4% berries concentration feed is an established optimal neuroprotective effect intake (Subash et al. 2014).

### 2.5 Estimation of Total Phenolic (TPC) and Flavonoid (TFC) Contents in Mulberry and Blueberry Fruits

#### 2.5.1 Sample Preparation

A 100 g of fresh berries samples were minced, and 5 g from the *M. alba* and blueberry were mixed with 25 mL of distilled water. The *M. macroura* was minced, and 5 g was mixed with 25 mL of distilled water. The mixtures were combined using the stirrer for 2 h. Then, the mixture was transferred to centrifuge tubes and was centrifuged at 6000 rpm for 20 min. The supernatant was separated from the precipitate and passed through Whatman filter paper. All the estimation procedures were carried out as described by Chandra et al. (2014).

#### 2.5.2 Estimation of TPC

TPCs of *M. alba*, *M. macroura*, and *S. mascatense* fruits were determined by the Folin–Ciocalteu method. Various concentrations of mulberry and blueberry extracts were mixed with 0.2 M Folin–Ciocalteu reagent and the reaction was neutralized by adding 7.5% (w/v) sodium carbonate (Na_2_CO_3_). This reaction mixture was incubated at room temperature for half an hour. A blue color was developed, and the absorbance was measured at 765 nm spectrophotometrically, with deionized water as blank. Gallic acid (GA) was used as a standard. The data were expressed as milligram gallic acid equivalents (GAEs) per gram of freeze‐drying powder. Finally, the data were converted to mg GAE/g dry‐solid basis, based on the moisture contents of powder and fresh fruit material.

#### 2.5.3 Estimation of TFC

The TFCs in the berries were estimated by aluminum chloride calorimetric method. Different concentrations of *M. alba*, *M. macroura*, and *S. mascatense* extracts and standard were prepared and mixed with 300 µL 5% (v/v) NaNO_2_, 300 µL 10% AlCl_3_. Following the mixing, 2 mL 1 M NaOH was added. Distilled water was used to make up the volume to 10 mL. Subsequently, the mixture was incubated at room temperature for 5 min. The absorbance was measured at 510 nm against the blank. The flavonoid contents were expressed as milligram catechin equivalents (CEQs) per gram of freeze‐dry powder. Finally, the data were converted to mg CEQ/g dry‐solid basis, based on the moisture contents of powder and fresh fruit material.

### 2.6 Antioxidant Biochemical Assays

The inhibition of lipid peroxidation was determined by the thiobarbituric acid reactive substances (TBARS) assay as a one measure of oxidative stress. Antioxidant capacity assay was estimated by the glutathione assay (GSH, GSSG, and total) to assess cellular redox status. Enzyme activity assays (superoxide dismutase [SOD], catalase [CAT], glutathione reductase [GR], and glutathione peroxidase [GPx]) were performed to measure the functional capacity of antioxidant enzymes like SOD, CAT, GR, and GPx in reducing oxidative stress utilizing kits and spectrophotometric techniques per manufacturers instruction (Ganapathy et al. 2020). The biological samples were the mice plasma. Typically, ∼0.5 mL of plasma was collected as terminal procedure. At sacrifice, the brain hippocampus samples were collected for WB analysis. The biochemical assays were conducted after the behavioral parameters experiments executed.

#### 2.6.1 TBARS Assay

The levels of TBARS were assessed according to Fraga et al. (1988). In the TBARS assay, malondialdehyde (MDA) and other TBARS were measured by their reactivity with thiobarbituric acid (TBA) in acidic conditions to generate a pink‐colored chromophore. Then, 0.5 mL of plasma was treated with 2.0 mL of TBA–TCA–HCl reagent and mixed thoroughly. The mixture was kept in a boiling water bath for 15 min. Then, the mixture was cooled, and the tubes were centrifuged for 10 min. The supernatant was collected and analyzed for TBARS assay. Standard solutions of 2–10 nmol were prepared and treated in the same manner. The absorbance of chromophore was read at 535 nm against the reagent blank using UV–Visible Spectrometer, and the values were expressed nmol/mL plasma.

#### 2.6.2 SOD Assay

The SOD assay was done using a kit (Catalog #K335‐100) from Bio Vision, USA. The kit contents are WST Solution, SOD Enzyme Solution, SOD Assay Buffer, and SOD Dilution Buffer. All the components were ready to use as supplied. A 20 µL of each sample was added to a separate 96‐well plate as well as blank 2. A 20 µL of distilled water was added to blank 1 and blank 3. A 200 µL of WST working solution was added to each well containing the samples and the blanks. A 20 µL of enzyme working solution was added to each well containing the samples and blank 1. A 20 µL of dilution buffer was added to the wells containing blank 2 and blank 3. Then, the plate was covered and incubated at 37°C for 20 min, and the absorbance was read at 450 nm using a Microplate reader Biochrom EZ read 400 (Cambridge, UK).

$$\mathrm{SOD}\;\mathrm{activity}\left(\mathrm{inhibition}\mathrm{rate}\%\right)=\left[\left(A_{\mathrm{blank}1}-A_{\mathrm{blank}3}\right)-\left(A_{\mathrm{sample}}-A_{\mathrm{blank}2}\right)/\left(A_{\mathrm{blank}1}-A_{\mathrm{blank}3}\right)\right]\times 100$$

The SOD activity was expressed as U/mL.

#### 2.6.3 Glutathione (GSH) Assay

The glutathione assay was carried out by using the analytical kit obtained from Bio Vision, USA Catalog (#K264‐100). All the samples were pre‐treated with 5 µL of 3% H_2_O_2_. Then, the samples were incubated at 25°C for 5 min following which the supernatant was centrifuged for 2 min at 13,000 × *g* at 4°C. The samples were analyzed for glutathione, total glutathione, and GSSG, according to the protocol given in the kit.

#### 2.6.4 GR and GPx Assays

The assay was carried out by using the analytical kit obtained from Bio Vision, USA (Catalog #K761‐200 and #K762‐100). All the reagents were supplied in the kit, and the experiment was carried out according to the protocol provided in the kit.

#### 2.6.5 Catalase (CAT) Assay

The assay was carried out by using the analytical kit obtained from Bio Vision, USA (Catalog # K773‐100). All the reagents were supplied in the kit, and the experiment was carried out according to the protocol provided in the kit.

### 2.7 Behavioral Parameters

For AlCl_3_‐treated mice, behavioral testing using the Morris water maze (MWM) and the T‐maze was implemented after a chronic administration period of 30 days.

#### 2.7.1 Morris Water Maze

The MWM was invented by Richard Morris, and this protocol helps researchers to understand the spatial learning and memory in animals (Vorhees and Williams 2014). It is a standard behavioral test for assessing hippocampus‐dependent spatial learning and memory in rodents. The animal's ability to discover the hidden platform was considered to assess the learning and memory with the help of cues in the maze. The maze consists of a spheroid tank 125 cm in diameter, 36 cm in height placed on top of a stand, and 20 cm above the floor. The maze was distributed equally into four quadrants (Q1, Q2, Q3, Q4) to create an imaginary “plus” (+) sign. Each quadrant was designated as SE, SW, NE, and NW. A platform with 10 cm^2^ diameter was placed in the Q3 throughout the training phase. The maze was filled with water until 1 cm above the platform. Water was made opaque/blurred with the help of a nontoxic material and maintained at 19–22°C to prevent hypothermia. Multiple protocols are available for MWM; the basic idea is animals should manage to reach the hidden platform by utilizing the available cues. The experiment was conducted in two phases—the training phase and the probe trial.

Animals were gently placed in a fixed quadrant (leaving position should not change throughout training and probe trial), the animal's head facing toward the wall of the maze. Animals were allowed to explore the maze and reach the hidden platform located in the third quadrant. The animals that could not reach the platform were guided with the help of a stick to the platform. Once the animal climbed the platform, they were allowed to explore for 15 s. The animals were taken out of the maze, wiped, dried, and then returned to the cage. Time spent by the animal in the target quadrant was assessed using a video tracking software called Any Maze. Animals were trained twice a day for 5 days. After learning for 5 days, the retention memory was tested on the sixth day, 24 h after the last day of training. During the probe, the trial platform was removed from the Q3 quadrant, and the animals were released from the origin, same as that of the training phase.

#### 2.7.2 T‐Maze

The T‐maze is used to assess the reference memory in mice; the test was performed according to Delarco et al. (2007). The T‐maze was filled with water (23°C ± 1°C). The main arm (100 cm × 20 cm × 40 cm) was connected to both short arms (45 cm × 20 cm × 40 cm), right and left. There were two manually operated sliding doors for both the short arms. The apparatus was equipped with a platform at the end of each arm and was submerged 1 cm below the water. The three trials of forced‐choice runs were carried out to assess the reference memory. A pair of the trials were incorporated with a forced run in which the animal was allowed to enter only one arm, where a submerged platform to escape from the water was present. After that a choice run was done, in which the animals were allowed to access both the arms, but the platform was placed in the arm opposite to the one which was not explored in the forced run. Hence, the animal learned to alternate to find the hidden platform during the choice run. If the animal chooses the arm in which the platform was absent, then the sliding door of that particular arm was closed, and the animal was kept there for 10 s. Then, the sliding door was opened for the animal to find the submerged platform, once the animal gets on top of the platform. Once the animal reached the platform in either forced or choice run, the sliding door was closed, and the animal was kept on the platform for 10 s. The animals were trained for 3 days, and assessment was performed on the next day.

### 2.8 WB Analysis

WB is a molecular biology technique used to detect specific proteins in a sample by separating them via gel electrophoresis, transferring them onto a membrane, and identifying the target protein using specific antibodies. WB is the standard, preferred method for determining Insulin Receptor Substrate 1 and 2 (IRS‐1 and ‐2) protein levels because it allows for specific identification, quantification, and analysis of these proteins in complex biological samples. It is crucial for studying insulin resistance, diabetes, and metabolic signaling because it reveals whether changes in signaling activity are due to altered protein expression or posttranslational modifications. Following euthanasia per Animal Ethical Committee approved protocol, the brains were collected, and the hippocampus was isolated. The tissues were lysed with a commercially available cocktail containing RIPA buffer and protease inhibitors (ThermoFisher). The total protein concentration was estimated using bicinchoninic acid protein assay (BCA) (Bio‐Rad Laboratories); the tissue lysate was stored at −80°C until use. About 20 µg of proteins were separated using 12% bis‐tris‐SDS–PAGE (Bio‐Rad) gel electrophoresis and electroblotted onto a PVDF (polyvinylidene fluoride) membrane. Following electroblotting, the membranes were blocked with non‐fat‐skimmed milk dissolved in TBST (Tris‐buffered saline‐Tween‐20) for 1 h following which the blots were incubated overnight with primary antibodies for IRS‐1 (1:1000) and IRS‐2 (1:1000) at 4°C. Thereafter, the blots were subjected to TBST washes (three washes for 5 min each at room temperature). Then, the blots were incubated with secondary antibodies (HRP‐conjugated anti‐mouse) for 1 h, followed by three TBST washes. The antigen–antibody complex formed will be detected using ECL detection reagents and imaged with the help of Uvitec Alliance Q9.

### 2.9 Statistics and Data Analysis

Data analysis was carried out by using graph pad prism version 5.0 (San Diego, USA) software. Multiple comparisons were performed by using one‐way ANOVA, and Tukey's multiple comparison test was used for *post hoc* comparisons. *p* value ≤0.05 was considered significant. Data were expressed as mean ± SEM.

## 3 Results

### 3.1 TPC and TFC of Mulberry and Blueberry Fruits

#### 3.1.1 Total Phenolic Content

**FIGURE 1:**
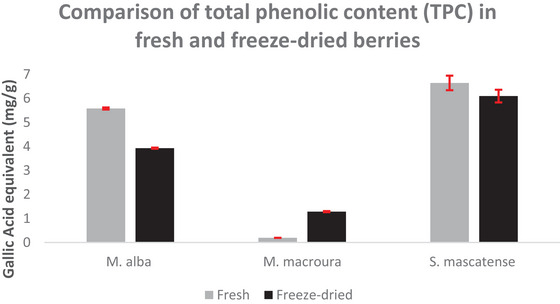
**Total phenolic content in fresh and freeze‐dried berries**. TPC is presented as mg of gallic acid equivalent/g of fresh or dry weight. All readings were taken in triplicate. The data are presented as mean ± SD.

The quantitative differences in the TPC were observed among the three berry varieties (*M. alba, M. macroura*, and *S. mascatense*) cultivated in Oman. The quantitative assay was performed on both the fresh and freeze‐dried berries to establish equivalency, as these fruits are seasonal with limited availability, in contrast to fresh fruit, the freeze‐dried powder form is more amenable to long‐term storage and relatively easier to mix with standard chow diet. Among the three berries, *S. mascatense* varieties had the highest concentration of TPC 6.642 ± 0.302 mg of GAE/g of fresh weight, whereas the lowest levels were seen in *M. macroura* (0.195 ± 0.001 mg of GAE/g of fresh weight). The TPC in the *M. alba* was found to be 5.578 ± 0.042 mg of GAE/g of fresh weight. In the case of the dried berries, the *S. mascatense* varieties contain the highest amount of TPC (6.095 ± 0.264 mg of GAE/g of dry weight). In contrast, *M. macroura* varieties had the lowest levels (1.285 ± 0.021 mg of GAE/g of dry weight) of TPC. The TPC in *M. alba* was found to be 3.926 ± 0.024 mg of GAE/g of dry weight (Figure 1).

#### 3.1.2 Total Flavonoid Content

**FIGURE 2:**
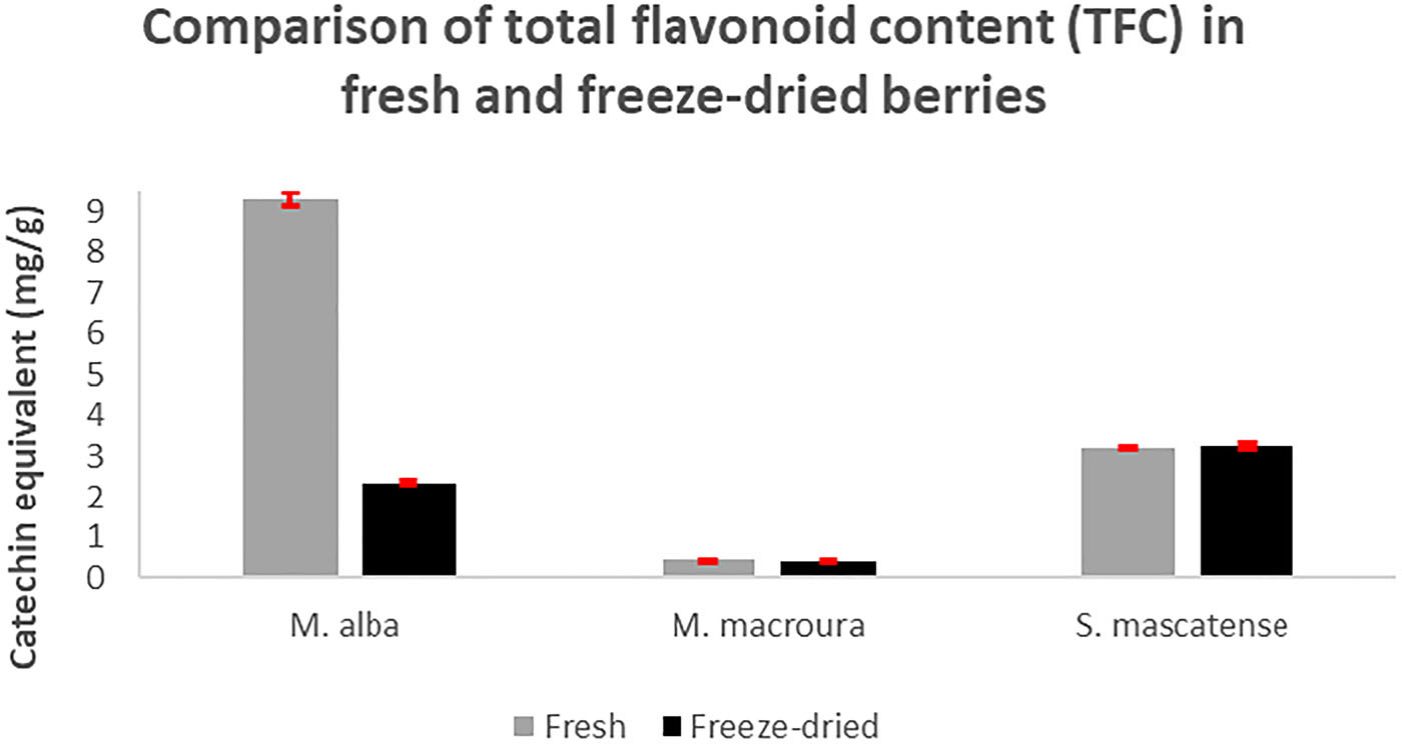
**Total flavonoid content in fresh and freeze‐dried berries**. TFC is presented as mg of catechin equivalents/g of fresh or dry weight. All readings were taken in triplicate. The data are presented as mean ± SD.

The TFC in the three varieties of the berries in both fresh and dried forms was assessed per rational mentioned above. *M. alba* was found to have the highest level of flavonoid content (9.306 ± 0.172 mg of CEQ/g of fresh weight), whereas the lowest levels were found in *M. macroura* (0.440 ± 0.001 mg of CEQ/g of fresh weight). TFC level in *S. mascatense* was found to be 3.218 ± 0.027 mg of CEQ/g of fresh weight. The dried berries of *S. mascatense* had the highest TFC content (3.279 ± 0.067 mg of CEQ/g of dry weight), whereas *M. macroura* had the lowest level (0.421 ± 0.009 mg of CEQ/g of dry weight). The TFC levels in *M. alba* were 2.361 ± 0.043 mg of CEQ/g of dry weight (Figure 2).

### 3.2 Supplementation With Berries Decreased the Elevated Lipid Peroxidation in AlCl_3_ Fed Mice

**FIGURE 3:**
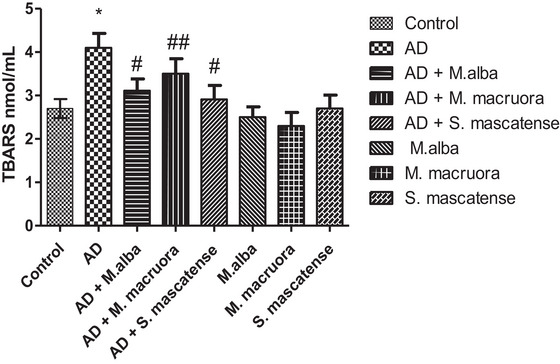
**TBARS levels determination in experimental mice**. Supplementation of diet with berries decreased lipid peroxidation in AD mice. AD, Alzheimer's disease; TBARS, thiobarbituric acid reactive substances.

Vehicle treated AlCl_3_ fed mice showed a significant increase in their lipid peroxidation compared to the normal control group (^*^ *p *˂ 0.05). Overall, groups supplemented with berries (groups 3, 4, and 5 *M. alba, M. macroura*, and *S. mascatense*, respectively) showed a significant reduction in their lipid peroxidation. Here, *M. macroura* treated mice showed a significant (^##^ *p *< 0.01) decrease in the lipid peroxidation value compared to the AD mice. Likewise, *M. alba and S. mascatense* treated mice had significantly (^#^ *p *< 0.05) reduced TBARS levels than AD mice. Moreover, groups supplemented with fruits alone, which functions as an internal control (groups 6, 7, 8 *M. alba, M. macroura*, and *S. mascatense*, respectively) showed nearly normal values of lipid peroxidation (Figure 3).

### 3.3 Berries Supplementation Improved the Decreased SOD Activity in AlCl_3_ Fed Mice

**FIGURE 4:**
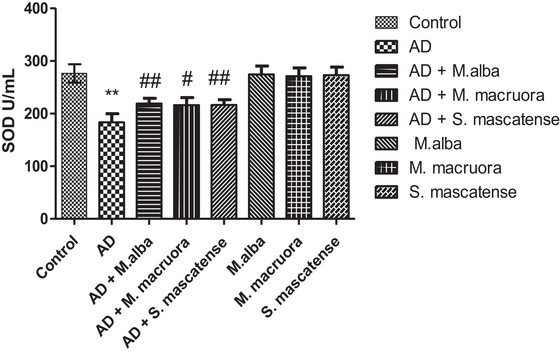
**SOD determination in experimental mice**. Berries supplementation improved superoxide dismutase (SOD) activity in AlCl_3_ fed mice. AD, Alzheimer's disease.

The vehicle‐treated AlCl_3_ fed mice showed a significant decrease in the SOD activity (^**^ *p *< 0.01), whereas the groups supplemented with berries (groups 3, 4, 5 *M. alba, M. macroura*, and *S. mascatense*, respectively) exhibited increased SOD levels (^#,##^ *p *< 0.01). The groups supplemented with berries alone did not show significant difference in their SOD levels compared to the normal group (Figure 4).

### 3.4 AlCl_3_ Administration Significantly Reduced the GSH Levels While the Berries Supplementation Reversed the Effect

**FIGURE 5:**
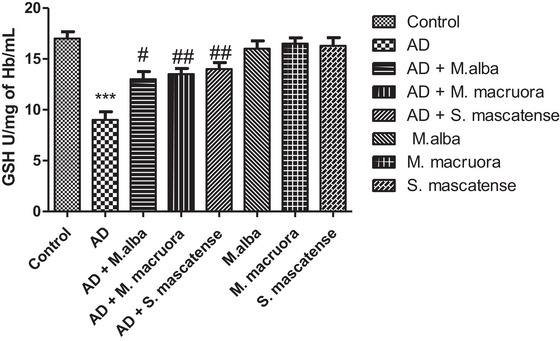
**Estimation of antioxidant capacity in experimental mice**. Glutathione assay showed lower GSH levels in AD mice; however, treatment with berries significantly improved the GSH levels. AD, Alzheimer's disease.

The vehicle‐treated AlCl_3_ fed mice showed a significant decrease in the GSH levels (^***^ *p *< 0.001). Overall, supplementation with berries showed a significant increase in the GSH levels compared to the vehicle‐treated group. Here, *M. alba* fed mice GSH activity improvement were noteworthy (^#^ *p *< 0.05), whereas *M. macroura* and *S. mascatense* fed mice GSH activity improvement were substantial (^##^ *p *< 0.01). Groups supplemented with berries alone showed normal levels of GSH (Figure 5).

### 3.5 AlCl_3_ Administration Depleted the GPx Levels; However, Supplementations With All the Berry Varieties Reversed the GPx Levels

**FIGURE 6:**
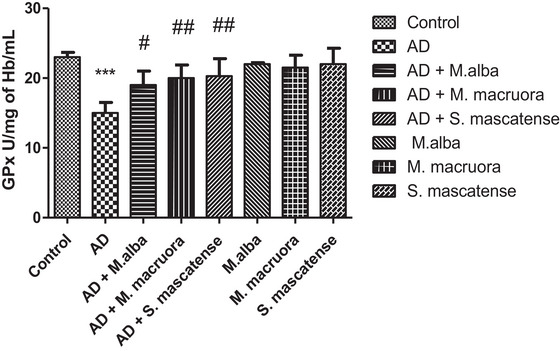
**GPx levels determination in the experimental groups**. GPx assay demonstrated declined GPx levels in AD mice, whereas mice treated with berries displayed increased GPx levels. AD, Alzheimer's disease; GPx, glutathione peroxidase.

Vehicle‐treated mice fed with AlCl_3_ showed a significant decline in their GPx levels (^***^ *p *< 0.001) compared to the normal controls. Overall, supplementation with berries significantly increased the GPx levels compared to the vehicle‐treated group. Here, treatment with *M. alba* (^#^ *p *< 0.05), whereas *M. macroura* and *S. mascatense* (^##^ *p *< 0.01) significantly displayed increased GPx levels compared to vehicle‐treated groups. Groups supplemented with berries alone showed the normal levels of GPx (Figure 6).

### 3.6 AlCl_3_ Administration Significantly Decreased CAT Levels; However, Treatment With Berries Escalated the Catalase Levels

**FIGURE 7:**
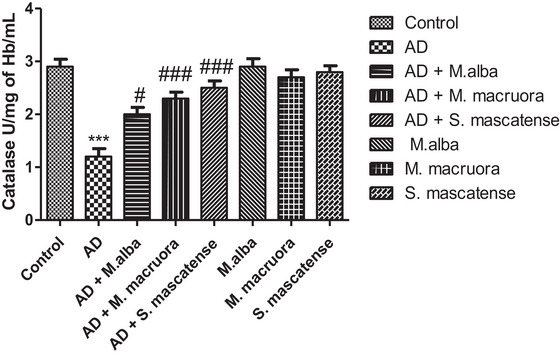
**CAT levels determination in the experimental groups**. CAT assay demonstrated declined CAT levels in AD mice, whereas mice treated with berries displayed increased CAT levels. AD, Alzheimer's disease.

AlCl_3_ administered vehicle‐treated mice showed a significant decline in the catalase levels (^***^ *p *< 0.001) compared to the vehicle alone treated group. On the other hand, treatment with berries mainly with *M. macroura* and *S. mascatense* significantly (^###^ *p *< 0.001) displayed increased CAT levels compared to the vehicle‐treated group. Groups supplemented with berries alone retained the normal levels of catalase (Figure 7).

### 3.7 Supplementation With Berries Showed an Improvement in Spatial Learning and Memory in AlCl_3_ Fed Mice

**FIGURE 8:**
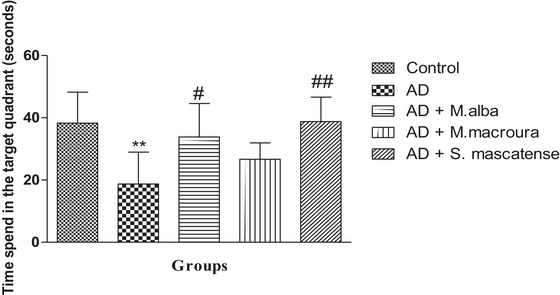
**Spatial learning and memory in MWM task**. AD mice exhibited significantly decreased time spent in the target quadrant, whereas supplementation specifically with *Morus alba* and *Sideroxylon mascatense* showed significant improvement in the time spent in the target quadrant when compared to positive control animals. AD, Alzheimer's disease.

Vehicle‐treated mice fed with AlCl_3_ showed a significant (^**^ *p *˂ 0.01) decrease in the time spent in the target quadrant during the MWM task compared to the normal group. Conversely, supplementation with *M. alba* significantly (^#^ *p *˂ 0.05) improved the time spent in the target quadrant. In addition, *S. mascatense* also showed a significant (^##^ *p *˂ 0.01) escalation in the time spent in the target quadrant compared to the positive control animals (Figure 8).

### 3.8 Berries Supplementation Attenuates Impaired Learning and Memory in T‐Maze in Response to AlCl_3_ Administration

**FIGURE 9:**
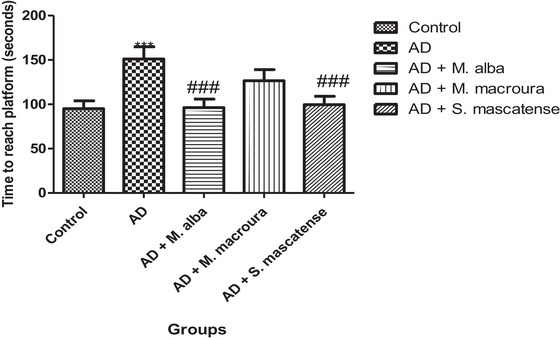
**Time taken to reach the platform (T‐maze test)**. AD mice treated with vehicle and AlCl_3_ took a significantly longer time to reach the platform compared to normal control, whereas supplementation specifically with *Morus alba* and *Sideroxylon mascatense* significantly minimized the time taken to reach the target platform. AD, Alzheimer's disease.

After the training phase, the time taken by the animals to reach the hidden platform was assessed. Vehicle treated mice fed with AlCl_3_ administration showed significant (^***^ *p *< 0.001) escalation in the time taken to reach the hidden platform compared to the normal animals. However, treatment with *M. alba* and *S. mascatense* varieties of berries showed a significant (^###^ *p *< 0.001) decrease in the time taken to reach the platform compared to the control animal. Surprisingly, treatment with *M. macroura* showed a nonsignificant reduction in the time taken to reach the hidden platform compared to the control animals (Figure 9).

### 3.9 Berries Supplementation Upregulated IRS1 and IRS2 Proteins in Hippocampal Neurons of the AlCl_3_ Fed Mice

**FIGURE 10:**
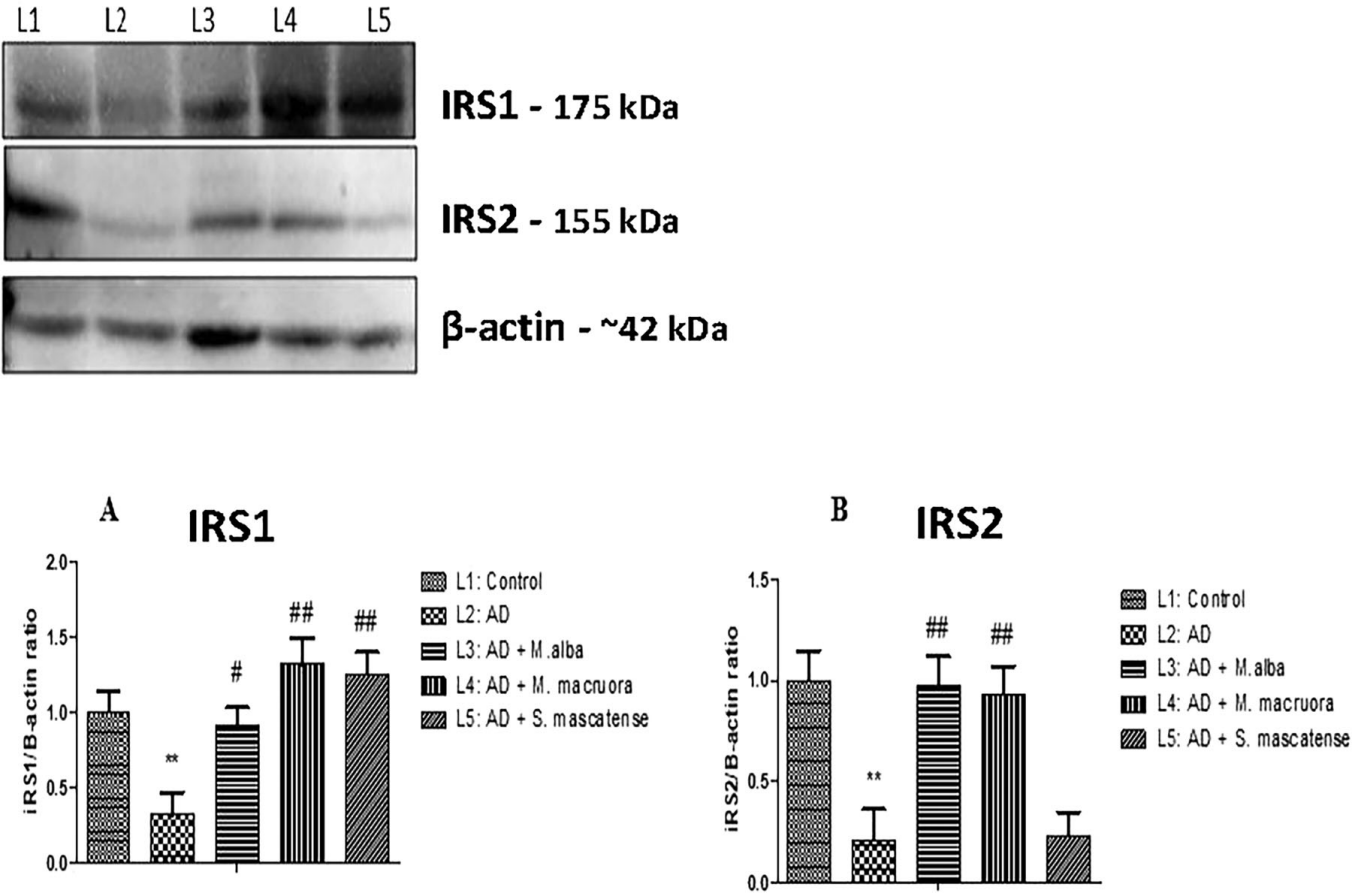
**Berries supplementation upregulated the IRS1 and IRS‐2 expression in AlCl_3_ fed mice**. Following euthanasia, the brains were dissected, and the hippocampus was isolated. After tissue processing, the expressions of IRS1 and IRS2 were detected by western blotting. (**A)** Quantification of IRS1/β‐actin. (**B)** Quantification of IRS2/β‐actin. Data are represented as mean ± SEM (*n* = 3) and represent three independent experiments. (^**^denotes *p *< 0.01 vs. vehicle, ^#^denotes *p *< 0.05, ^##^denotes *p *< 0.01 vs. AlCl_3_ fed positive control).IRS1 (insulin receptor substrate 1) observed Mwt is ∼160–185 kDa, whereas the predicted is ∼131–132 kDa; it frequently migrates at a higher weight on gels due to extensive serine phosphorylation. IRS2 (insulin receptor substrate 2) observed Mwt is ∼150–190 kDa, whereas the predicted is 137–138 kDa. β‐actin is ∼42 kDa. It is a highly conserved protein and is consistently used as a loading control at this Mwt. AD, Alzheimer's disease.

Insulin receptor signaling (IRS) is crucial for various neuronal activities, including learning and memory. As shown in Figure 10, AlCl_3_ administration significantly downregulated both the IRS‐1 and IRS‐2 expression. However, supplementation with berries improved insulin signaling. Treatment with *M. alba* and *M. macroura* significantly upregulated both the IRS‐1 and IRS‐2 levels. Supplementation with *S. mascatense* improved the IRS‐1 signaling; however, it did not show a positive effect on IRS‐2 signaling (Figure 10).

## 4 Discussion

AD is a progressive chronic neurodegenerative disorder characterized by age‐dependent dementia. The major pathological hallmarks of the extracellular aggregation of Aβ peptides, tau proteins, and the neurofibrillary tangles (Ballatore et al. 2007; Bharathi and Thenmozhi 2018). Earlier studies showed that chronic administration of AlCl_3_ escalated the aluminum concentration in both hippocampus and cortex (Sakamoto et al. 2004; Abubakar et al. 2004). Moreover, aluminum gets accumulated in various regions of the brain, mainly in the hippocampus (Kaur and Gill 2006). Thus, aluminum increases the Aβ levels and other enzymes involved in the production of the Aβ (Hampel et al. 2021). Increased levels of Aβ lead to cognitive deficits and memory impairment (Lei et al. 2020). AlCl_3_ also worsens the oxidative status of the brain. Oxidative stress is regarded as one of the major causes of AD pathology (Baldeiras et al. 2008; Ferreiro et al. 2012; Greilberger et al. 2008). There are various sources of the free radical surge in AD‐like mitochondrial dysfunction, accumulation of Aβ, NADPH, and inflammation (Padurariu et al. 2010). Existing evidence confirmed mitochondrial dysfunction following AlCl_3_ administration (Vučetić‐Arsić et al. 2013).

Elevated levels of free radicals lead to lipid peroxidation, which in turn triggers the events that urge neurodegeneration and AD. The end products of lipid peroxidation, like 4‐hydroxy‐2‐nonenal (4‐HNE) and MDA, are neurotoxic in nature, cause neuronal membrane disruption, and also posttranslational changes in the key proteins that are crucial in the pathogenesis of AD (Sultana et al. 2013). The results of the present study also showed similar effects with TBARS levels following AlCl_3_ administration. There was a significant increase in the TBARS levels in the vehicle‐treated animals fed with AlCl_3_, whereas the supplementation with berries significantly decreased the TBARS levels. Both *M. alba* and *S. mascatense* variety of berries showed prominent levels of polyphenols and flavonoids (Figures 1 and 2). As the above berry types are rich in polyphenols and flavonoids, they showed better activity in the TBARS levels (Figure 3). Earlier studies revealed that varieties of blueberries and mulberries have potent antioxidant properties (Sweeney et al. 2002; Arfan et al. 2012).

Lower levels of SOD, GSH, and GPx enzymes lead to oxidative damage in the prominent regions of the brain (Figures 4‐6). The above enzymes possess free radical scavenging property, and they detoxify the secondary metabolites formed following lipid peroxidation. Earlier studies showed that compromised oxidative status and surge in free radical associated neuronal damage aggravate the AD pathology (Saharan and Mandal 2014). Abdallah et al. (2014) showed significant decrease in the SOD, GSH, and GPx levels in the brain following AlCl_3_ administration. The results of the present study also showed similar results with SOD, GSH, and GPx levels including CAT, following AlCl_3_ administration. However, supplementation with berries of *M. alba, M. macroura*, and *S. mascatense* showed a significant increase in the SOD, GSH, GPx and CAT levels. The results were consistent with the previous studies of Çoban et al., who revealed that treatment with blueberries improved SOD, GSH, and GPx levels in rat brain. In addition, blueberries alleviated oxidative stress‐induced histopathological abnormalities in the rat brain (Çoban et al. 2015).

Several studies revealed the deleterious effect of AlCl_3_ administration on cognitive performance examined with the help of various cognitive tasks (Hosseini‐Sharifabad et al. 2020). In the present study, the authors performed MWM and T‐maze task to assess the cognitive performance in the animals. In MWM task, the authors examined the time spent in the target quadrant, where the escape platform was placed during the training phase. In the vehicle‐treated group fed with AlCl_3_, the animals showed a significant decrease in the time spent in the target quadrant; however, supplementation with berries of both *M. alba* and *S. mascatense* has improved the time spent in the target quadrant. Likewise, similar results were observed in the T‐maze task on supplementation with both *M. alba* and *S. mascatense* which decreased the time taken to reach the platform.

IR plays a crucial role in various neuronal activities, including learning and memory. Improved cognitive performance mediated through IRs was studied in both humans and animals (Kern et al. 1999; Park et al. 2000). However, impaired insulin receptor signaling is associated with the development of neurodegenerative disorders like AD (Watson and Craft 2003; Gasparini and Xu 2003). Many studies revealed that insulin regulated the Aβ levels (Mullins et al. 2017). A recent study suggested that insulin accelerated the movement of Aβ from Golgi apparatus and trans‐Golgi network to the plasma membrane (Gasparini et al. 2001). In addition, insulin promoted Aβ degradation through insulin degrading enzyme (IDE). Several studies reported that IDE played a vital role in the clearance of Aβ (Kurochkin and Goto 1994; McDermott and Gibson 1997; Baranello et al. 2015). Thus, normal functioning of the insulin pathway is essential to alleviate the accumulation of the toxic metabolites in the AD pathology. The WB analysis revealed a significant downregulation of IRs following the AlCl_3_ administration. However, treatment with *M. alba* and *S. mascatense* upregulated the IRs. The present study results were consistent with Seymour et al. (2011), where treatment with blueberries helped overcome insulin resistance.

Furthermore, a recent publication on the MIND diet adherence (MIND is an acronym for “Mediterranean‐DASH Intervention for Neurodegenerative Delay” where berries are a major component of the brain health food group) not only improved cognitive impairment and decline but also reduced systemic inflammation, facilitated weight loss, enhanced the health of the microbiome, ameliorated insulin resistance, lowered elevated blood lipids, and slowed atherogenesis (Sawyer et al. 2024). A prior systematic review analysis (Bonyadi et al. 2022) concluded that berries consumption may be endorsed as part of a healthy dietary approach to preventing cognitive decline among the elderly. Collective evidence (in vitro models, animal models, observational studies, and clinical research) suggests that the phytochemicals anthocyanins make the greatest impact on health functionality (anti‐inflammatory and antioxidant activities) as they reduce risk of cardiovascular diseases and type 2 diabetes, promote microflora and weight maintenance, and offer neuroprotection including neurodegenerative conditions thus decreasing the risk of cognitive decline (Kalt et al. 2020).

It is worthy to state the experimental limitations in studies on berry varieties and cognitive decline, which may constrain evidence strength and generalizability. Typically, the most cited is sample size and statistical power, and possibly species and gender bias, though our study has been designed to many others that have been published. Conceivably, another limitation the heterogeneity within the berry variety or residual confounders like mouse microbiota that are very difficult to control. Additionally, berry phytocompounds bioavailability and pharmacokinetics may reduce effective brain concentrations and therapeutic efficacy. There is a need for long‐term validation studies to mitigate inconsistencies or contradictions. Finally, one could always question animal models (reproducibility and translatability) and their human relevance. Despite these limitations there is tremendous cumulative amount of evidence in the literature that demonstrates the valuable health benefits of berries fruits/extract and their neuroprotection function including mitigating cognitive decline.

## 5 Conclusions

This study demonstrates that supplementation with locally grown berries, specifically *M. alba* and *S. mascatense*, significantly improved cognitive performance in the MWM and T‐maze tasks. Their potent antioxidant properties helped mitigate insulin resistance and oxidative stress induced by AlCl_3_ administration, suggesting a strong neuroprotective potential. Given the high prevalence of both AD and T2DM in many populations, particularly those with increasing rates of metabolic disorders like the Gulf region, these findings warrant further investigation.

The demonstrated ability of these berries to restore IR signaling and combat oxidative stress positions them as promising candidates for nutraceutical development. Considering the well‐established link between impaired glucose metabolism and AD, incorporating these berries into dietary interventions may offer a dual therapeutic approach—targeting both insulin resistance and neurodegeneration. Future clinical studies should explore berry‐based interventions, especially in regions like the Middle East, where these fruits are culturally significant and widely available.

## Author Contributions

Sawsan G. A. A. Mohammed, Reem Al Alawi, and Shadhan Al‐Siyabi performed research and collected information, and generated short write‐ups. Sawsan G. A. A. Mohammed and M. Walid Qoronfleh provided research insight, content examination, and supported in numerous aspects during the manuscript development process. M. Walid Qoronfleh contributed to conceptual work, framework, final draft write‐up, critical reading, and editing. All authors read and approved the final manuscript.

## Funding

The authors have nothing to report.

## Ethics Statement

This research involves experimental animals. Animal use complied with institutional, national, and international guidelines including the Sultan Qaboos University. The study protocol was approved by Sultan Qaboos University (SQU) Animal Ethical Committee (SQU/AEC/2016‐17/5).

## Consent

The authors have nothing to report.

## Conflicts of Interest

The authors declare no conflicts of interest.

## Data Availability

All data generated or analyzed during this study are included in this published article. The data that support the findings of this study are available from the corresponding author upon reasonable request.
